# Induction of Liver Size Reduction in Zebrafish Larvae by the Emerging Synthetic Cannabinoid 4F-MDMB-BINACA and Its Impact on Drug Metabolism

**DOI:** 10.3390/molecules27041290

**Published:** 2022-02-15

**Authors:** Yu Mi Park, Charlotte Dahlem, Markus R. Meyer, Alexandra K. Kiemer, Rolf Müller, Jennifer Herrmann

**Affiliations:** 1 Helmholtz Centre for Infection Research, Helmholtz Institute for Pharmaceutical Research Saarland (HIPS), Campus E8 1, Saarland University, 66123 Saarbrücken, Germany; yu-mi.park@helmholtz-hips.de; 2 Environmental Safety Group, Korea Institute of Science and Technology (KIST) Europe, 66123 Saarbrücken, Germany; 3 Department of Pharmacy, Saarland University, 66123 Saarbrücken, Germany; 4 Department of Pharmacy, Pharmaceutical Biology, Campus C2 3, Saarland University, 66123 Saarbrücken, Germany; charlotte.dahlem@uni-saarland.de (C.D.); pharm.bio.kiemer@mx.uni-saarland.de (A.K.K.); 5 Center for Molecular Signaling (PZMS), Institute of Experimental and Clinical Pharmacology and Toxicology, Department of Experimental and Clinical Toxicology, Saarland University, 66421 Homburg, Germany; m.r.meyer@mx.uni-saarland.de; 6 German Center for Infection Research (DZIF), 38124 Braunschweig, Germany

**Keywords:** zebrafish larvae model, drug metabolism, cannabinoid receptors (CB1 and CB2), mass spectrometry imaging (MSI), hepatotoxicity, microinjection, morpholino oligonucleotides, synthetic cannabinoid (SC), methyl 2-[1-(4-fluorobutyl)-1*H*-indazole-3-carboxamido]-3,3-dimethylbutanoate (4F-MDMB-BINACA), methyl 2-(1-(5-fluoropentyl)-1*H*-pyrrolo[2,3-b]pyridine-3-carboxamido)-3,3-dimethylbutanoate (7′*N*-5F-ADB)

## Abstract

Zebrafish (ZF; *Danio rerio*) larvae have become a popular in vivo model in drug metabolism studies. Here, we investigated the metabolism of methyl 2-[1-(4-fluorobutyl)-1*H*-indazole-3-carboxamido]-3,3-dimethylbutanoate (4F-MDMB-BINACA) in ZF larvae after direct administration of the cannabinoid via microinjection, and we visualized the spatial distributions of the parent compound and its metabolites by mass spectrometry imaging (MSI). Furthermore, using genetically modified ZF larvae, the role of cannabinoid receptor type 1 (CB1) and type 2 (CB2) on drug metabolism was studied. Receptor-deficient ZF mutant larvae were created using morpholino oligonucleotides (MOs), and CB2-deficiency had a critical impact on liver development of ZF larva, leading to a significant reduction of liver size. A similar phenotype was observed when treating wild-type ZF larvae with 4F-MDMB-BINACA. Thus, we reasoned that the cannabinoid-induced impaired liver development might also influence its metabolic function. Studying the metabolism of two synthetic cannabinoids, 4F-MDMB-BINACA and methyl 2-(1-(5-fluoropentyl)-1*H*-pyrrolo[2,3-b]pyridine-3-carboxamido)-3,3-dimethylbutanoate (7′*N*-5F-ADB), revealed important insights into the in vivo metabolism of these compounds and the role of cannabinoid receptor binding.

## 1. Introduction

In drug discovery and development, many non-clinical studies are performed using animal models to investigate pharmacokinetics (PK) and bioavailability, non-clinical toxicology, and efficacy of new drug candidates [1]. During these early phases, 40% to 80% of compounds fail, and their further development is stopped mainly due to safety concerns and/or insufficient PK properties [1,2,3,4]. In order to reduce the number of animal experiments in pre-clinical testing and increase the rate of success in such models, many alternative models are being developed [1,2,3]. Zebrafish (*Danio rerio*; ZF) has become an important pre-clinical in vivo vertebrate model, which is widely applied in drug discovery to study the pharmacology of new drug candidates [5,6,7,8,9,10]. Eight small molecules which were initially discovered in ZF models have proceeded into clinical trials during the past decade [1,11,12,13,14,15,16,17,18], demonstrating that the use of ZF models can contribute to successful translation [1,18]. Over the past few years, the ZF model has been increasingly used not only in functional and safety studies but also in drug metabolism studies, and its reproducibility and high coverage of human metabolites have been demonstrated [19]. Numerous studies have been published on ZF xenobiotic metabolites [19,20,21,22,23,24], and importantly, several new psychoactive substances (NPSs) and their phase I and phase II metabolites in ZF have been evaluated and were found to well correlate with human metabolism [25,26,27,28].

**Figure 1:**
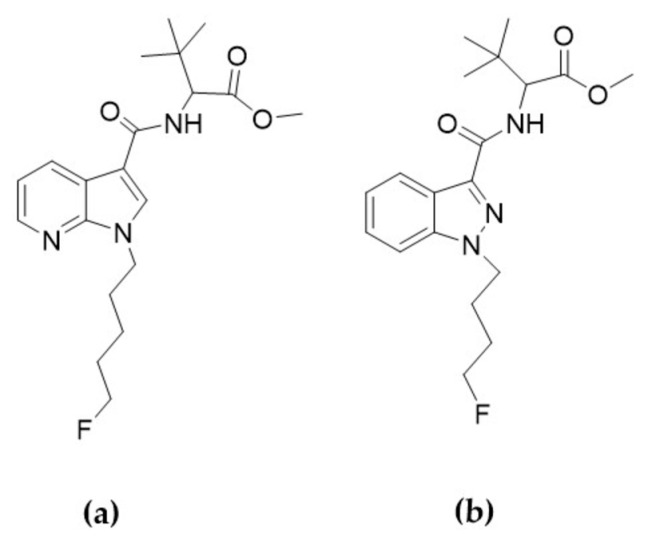
Chemical structures of 7′*N*-5F-ADB (**a**) and 4F-MDMB-BINACA (**b**).

In our previous study [28], we assessed various administration routes into ZF larvae, and an authentic spectrum of metabolites of the synthetic cannabinoid (SC) methyl 2-(1-(5-fluoropentyl)-1*H*-pyrrolo[2,3-b]pyridine-3-carboxamido)-3,3-dimethylbutanoate (7′*N*-5F-ADB; Figure 1a) was obtained in ZF larvae. Using mass spectrometry imaging (MSI), the spatial distribution pattern in ZF larvae of 7′*N*-5F-ADB and its metabolites was studied and revealed the impact of different administration routes on in vivo distribution and drug metabolism.

SCs are investigated as pharmacological probes to study the endocannabinoid system (ECS), and they show some pharmaceutical potential, e.g., in the treatment of inflammatory diseases and in cancer pain management [19,29,30]. However, new emerging SCs are also potentially harmful psychoactive drugs of abuse, and they pose a severe threat to human health with numerous reported associated fatalities [31]. Detailed information on their metabolic fate and a mechanistic understanding is almost entirely lacking due to the rapid generation of new derivatives and the illegal distribution of these new SCs.

In this study, we aimed to investigate the metabolism of a newly emerging SC, methyl 2-[1-(4-fluorobutyl)-1*H*-indazole-3-carboxamido]-3,3-dimethylbutanoate (4F-MDMB-BINACA, Figure 1b). 4F-MDMB-BINACA was chosen for this study because it is an emerging and highly potent new SC for which a high number of seizure cases were reported in 2019 by the European Union Early Warning System [31,32]. Therefore, 4F-MDMB-BINACA has been controlled under Schedule II of the Convention on Psychotropic Substances of 1971, designed as a United Nations treaty, which came into force on 3 November 2020 [33,34].

Here, we relied on direct administration routes into the ZF larvae, basically following the optimized protocol of our previous study [28]. The detected ZF metabolites were then compared to recently published human metabolites [27,32,34,35]. In addition, we explored the circulation/distribution of 4F-MDMB-BINACA inside the ZF larval body following microinjection of the SCs into internal organs (caudal vein, heart ventricle, and hindbrain) and visualized molecular spatial images obtained from MSI. Furthermore, 4F-MDMB-BINACA was described as a potent agonist of cannabinoid receptor type 1 (CB1) [31,34,36]. In ZF larvae, it was shown that loss of CB1 and cannabinoid receptor type 2 (CB2) lead to a significant reduction of liver size, impaired hepatocyte proliferation, and reduced liver gene expression [37,38,39]. In turn, this liver pathophysiologic effect could impact the ZF metabolism of SCs since phase I and phase II metabolic enzymes are expressed in this compartment. We then studied the correlation between drug metabolism and non-functional CB1 and CB2. To achieve this, we utilized morpholino oligonucleotides (MO) as an antisense gene-knockdown tool, which is widely and successfully used for, e.g., modeling human diseases in ZF [40,41,42]. The metabolic profiles of 4F-MDMB-BINACA and 7′*N*-5F-ADB (Figure 1) in ZF larvae mutants experiencing gene knock-down of either CB1 or CB2 were explored and compared to findings from our earlier studies [25,27,28].

## 2. Results and Discussion

### 2.1. Zebrafish Larvae Produce an Authentic Spectrum of 4F-MDMB-BINACA Metabolites

In our previous study, we optimized the administration route for 7′*N*-5F-ADB into ZF larvae to study the SCs metabolism [28]. This was the first comprehensive evaluation of the impact of compound administration (waterborne exposure vs. microinjection into different organs) on the efficacy of drug metabolism. In the case of the studied SC, we could reveal that only one metabolite can be detected when the drug was microinjected into the yolk sac of the ZF larvae. In contrast, a significantly higher number of metabolites could be detected by adding 7′*N*-5F-ADB into ZF larvae medium or microinjecting the SC into vital organs. Thus, owing to the presumed similar physico-chemical properties of 7′*N*-5F-ADB and 4F-MDMB-BINACA (cp. Section 2.3), we assessed ZF larvae metabolism of the latter following microinjection of the drug into vital organs such as the caudal vein, heart ventricle, and hindbrain.

Wagmann et al. [27] studied the metabolism of five NPSs, including 4F-MDMB-BINACA, and compared the metabolite spectra found in ZF larvae (waterborne exposure), in vitro models (pooled human liver S9 fraction and HepaRG cells), and one authentic human plasma sample. In total, 14 phase I and four phase II metabolites were detected in the ZF larvae, and ZF larvae produced the highest number of 4F-MDMB-BINACA (and four other NPSs) metabolites among all investigated models. Here, we further investigated the metabolism of 4F-MDMB-BINACA in ZF larvae using direct administration routes, with the aim to deepen the knowledge on human metabolism of the NPS. For this, we also compared our findings to recently published studies of human biosamples (blood and urine), which were screened for 4F-MDMB-BINACA and its metabolites from 2019 to 2021 as part of routine toxicology [27,34,35] or forensic casework [32].

Table 1 summarizes phase I and phase II metabolite data from microinjected ZF larvae investigated in this study, and the integrated data of human screening results reported from other studies were added along [27,32,34,35]. More detailed information on the human screening data surveyed is provided in Supplementary Table S1, which also provides the metabolic reactions and exact masses of the detected metabolites. The structure elucidations of these detected metabolites based on their MS^2^ data are reported elsewhere [27].

While combining human data, we found that these studies show some deviations with respect to metabolite detection, especially in urine samples, as the number of reported metabolites varies from three to twelve detected, depending on the studied sample. This discrepancy is possibly caused by the lack of homogeneity and diversity in random sampling, where no medical and personal information on the patients is available. Thus, the ‘integrated human samples’ data of Table 1 represent abundant metabolites of 4F-MDMB-BINACA, which were detected at least twice in each of the samples. In short, out of the 26 metabolites identified from three different models in our previous study [27], nine metabolites were observed in human urine samples and three metabolites in blood samples (Table 1 and Supplementary Table S1).

**Figure 2:**
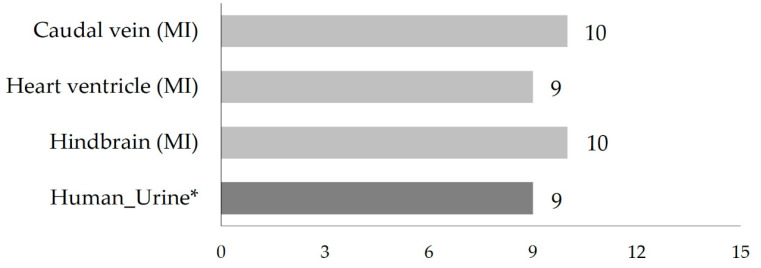
Total number of detected 4F-MDMB-BINACA metabolites following administration of the SC into ZF larvae through different microinjection routes. All results from ZF larvae in this study are represented as the mean value of peak numbers from triplicates of 30 pooled larvae. * Human urine data represent published integrated data of metabolites that were detected at least twice in all studied urine samples [27,32,34,35]. (MI: microinjection).

Upon microinjection of 4F-MDMB-BINACA into the caudal vein and hindbrain of ZF larvae, ten metabolites were detected. Similarly, following microinjection into the heart ventricle, nine metabolites were found, whereas the previously found M2 metabolite (lactone formation in combination with *N*-dealkylation) was not detected (Table 1 and Figure 2). Two metabolites, M20 (hydroxylation of the indazole part in combination with sulfation) and M25 (hydroxylation of the tertiary butyl part in combination with glucuronidation isomer 1), out of seven phase II metabolites, were produced in all microinjected ZF larvae. In human biosamples, there was no detection of phase II metabolites except M22 (ester hydrolysis in combination with glucuronidation) detected only once from urine samples [27].

M2 and M12 (lactone formation in combination with hydroxylation of the tertiary butyl part), which originate from M6 (lactone formation), were uniquely detected in ZF larvae but not in human samples (Table 1 and Supplementary Table S1). Conversely, M6 is the second most abundant human metabolite, and it was also detected in all investigated ZF larvae samples. The most abundant metabolite in human urine and blood samples, M8 (ester hydrolysis), was not detected in the microinjected ZF larvae. M3 (ester hydrolysis in combination with *N*-dealkylation), M10 (ester hydrolysis in combination with oxidative defluorination and oxidation to carboxylic acid), M11 (oxidative defluorination), and M15 (oxidative defluorination in combination with oxidation to carboxylic acid) were observed in both microinjected ZF larvae and integrated urine samples. M8 is metabolized to M7 and then to M10, however, in the microinjected larvae, only M10 as the latest metabolite was found. In contrast, M4 (*N*-dealkylation) was commonly observed in the ZF larvae and integrated blood samples, but it was not found in human urine samples (Table 1 and Supplementary Table S1). Based on the detection pattern of these four metabolites (M2, M6, M10, and M12), we conclude that ZF larvae in the chosen setup display a faster metabolism than found in humans.

**Figure 3:**
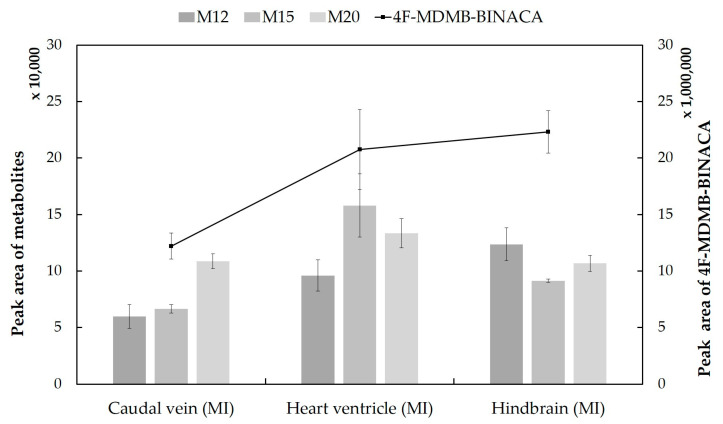
Detection profiles of the parent compound 4F-MDMB-BINACA and the three most abundant metabolites in microinjected ZF larvae (caudal vein, heart ventricle, and hindbrain). The most abundant metabolites (M12, M15, and M20) were formed by lactone formation, oxidative defluorination, and hydroxylation, respectively. The line chart represents the relative abundance of the parent compound, and three gray-colored clustered columns stand for its major metabolites. All data are displayed as mean ± standard deviation (SD) (n = 3).

Assessing the mutual comparability between ZF larvae and human biosamples, the metabolism of the NPS in microinjected ZF larvae displays a high similarity, with a 67% match rate to integrated human blood samples and a 56% match rate to integrated human urine samples, respectively. In the microinjected ZF larvae, the three most abundant metabolites of 4F-MDMB-BINACA were M12, M15 (oxidative defluorination in combination with oxidation to carboxylic acid), and M20 (hydroxylation of the indazole part in combination with sulfation). The parent compound, 4F-MDMB-BINACA, still existed in the injected larvae, and it was the most abundant peak (Table 1 and Figure 3).

Moreover, comparing the relative abundance of the three major ZF metabolites, their peak intensities varied depending on the site of microinjection. M20 was most abundant after caudal vein injection, M15 after heart ventricle injection, and M12 after injecting 4F-MDMB-BINACA into the hindbrain (Figure 3). The peak area of M20 was similar in all three microinjected ZF samples. However, the observed differences were mostly below statistical significance with *p* > 0.05. Overall, peak intensities of the parent compound and its three major metabolites were lowest after injection into the ZF caudal vein, and this finding was also confirmed for the three minor metabolites M4, M11, and M25 (Supplementary Figure S1). This result can possibly be explained by the fast circulation of the parent compound as soon as it is injected into the caudal vein. This, in turn, might cause fast metabolism and excretion. However, the overall metabolite pattern seems to be barely influenced by the target organ of microinjection as direct administration routes for 4F-MDMB-BINACA (Table 1 and Figure 2). In contrast to the previously studied cannabinoid 7′*N*-5F-ADB, direct injection of 4F-MDMB-BINACA into ZF larvae did not increase the number of detected metabolites compared to waterborne exposure [28].

**Figure 4:**
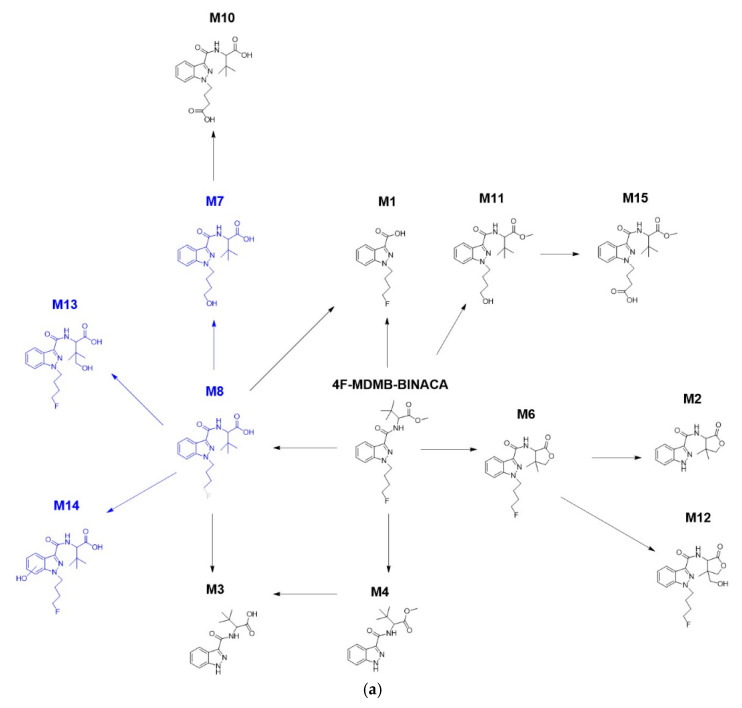

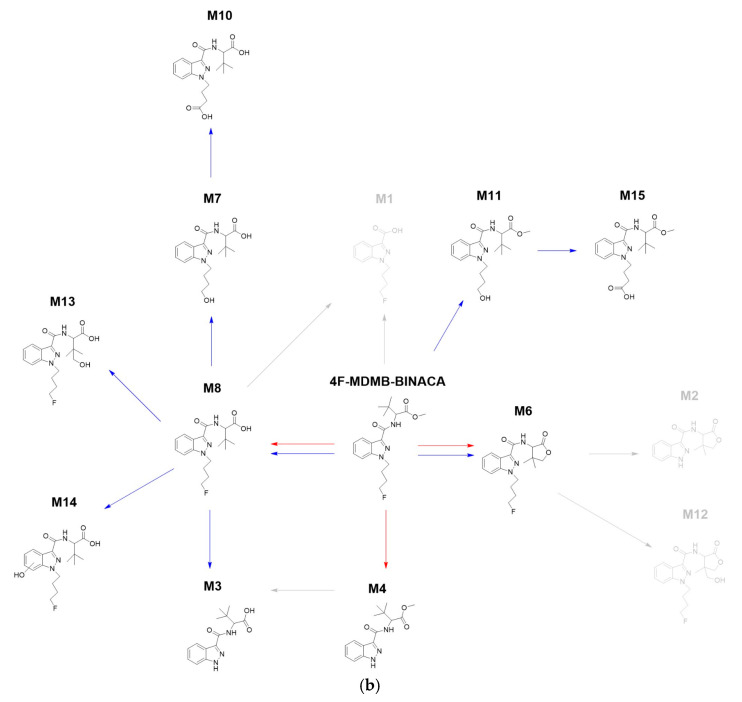
The main phase I metabolic pathway of 4F-MDMB-BINACA based on findings in the ZF larvae model (**a**) and from human screening (**b**). In panel (**b**), red arrows indicate results from human blood samples, and blue arrows indicate results from human urine samples. Metabolites in blue represent molecules not detected in the respective model. Four metabolites (M7, M8, M13, and M14) were not detected in the microinjected ZF larvae despite the detection of M3 and M10 as their following sequential metabolites. In contrast, terminal metabolites M1, M2, and M12 and a cross-connection between M3 and M4 were not found in human samples.

The main phase I metabolic pathways of 4F-MDMB-BINACA were reconstructed by complementing the metabolites detected in ZF larvae to integrated human biosamples [27,32,34,35], which contained M1 found by MALDI-MSI analysis (see Section 2.2). Conversely, the pathways for phase II metabolism were not constructed because the overall experimental metabolite detection in all investigated models was insufficient, with only two metabolites found in the microinjected ZF larvae (M20, M25) and one metabolite (M22) found in human biosamples. In the microinjected ZF larvae, four minor metabolites (M7, M8, M13, and M14) in the left panel of the phase I metabolic pathway were not produced (Figure 4a), whereas three other metabolites (M1, M2, and M12) and the cross-connection between M3 and M4 in the right panel were absent in human urine samples (Figure 4b). In human blood samples, only three primary metabolites (M4, M6, and M8) were found at the center of the pathway.

M3 and M10 were detected in the microinjected ZF larvae despite the non-detection of two intermediates (M7 and M8). Among all detected metabolites, M6, formed by lactone formation, is a reliable metabolic biomarker as it was found with high abundance in the ZF larvae model as well as in all previously investigated human biosamples (Table 1 and Supplementary Table S1). M6 was previously recommended as a target for toxicological screening in two studies [27,35], and we could confirm its high peak intensity detection in the ZF larva model. Furthermore, M8 also seems to be another metabolic biomarker in humans since it is predominant in all human urine and blood samples (Supplementary Table S1). Taken together, we were able to construct the human metabolic phase I pathway of 4F-MDMB-BINACA by combining phase I metabolites from ZF larvae with already described human metabolites.

### 2.2. Spatial Distribution of 4F-MDMB-BINACA and Its Metabolites in Zebrafish Larvae

MSI is a powerful tool and label-free technology that enables visualization of the spatial distribution of molecules in biological specimens in a single experiment [43,44,45,46,47]. In combination with a high-resolution mass spectrometer such as Fourier-transform ion cyclotron resonance (FT-ICR), MSI can depict the localization of molecules, which can be identified with high confidence. Moreover, MSI mapping of molecules of interest (e.g., drugs, metabolites, biomarkers) in biological tissues is critical for understanding their pharmacology [44,45]. In recent years, MALDI-MSI is becoming a more established tool in clinical practice and the pharmaceutical industry [45,46,47]. However, its application to ZF larvae is still limited, mainly due to challenging and critical steps of sample preparation. Nevertheless, we were previously able to generate spatial distribution images of 7′*N*-5F-ADB metabolites in ZF larvae by MALDI-FT-ICR [28].

In this study, we used MALDI-MSI to study the distribution of 4F-MDMB-BINACA in the ZF larval bodies and investigated how spatial MS images of molecules can be utilized to provide reliable metabolism information along with LC-HRMS/MS data.

**Figure 5:**
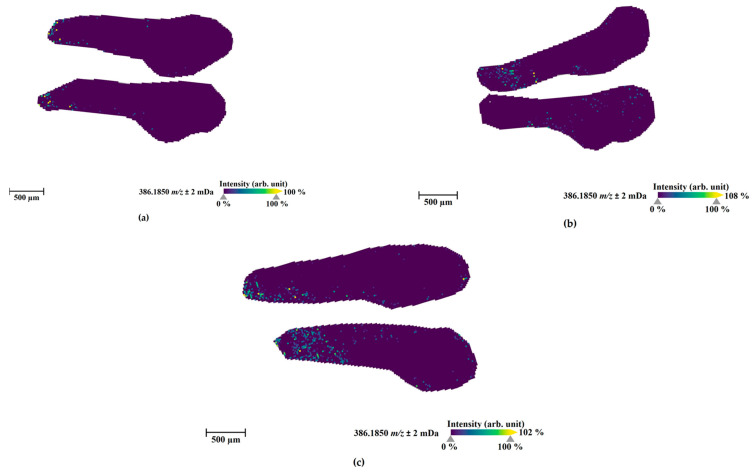
MALDI-MS images of the parent compound (4F-MDMB-BINACA, sodium adduct, m/z 386.1850) in zebrafish larvae at 4 days post-fertilization (dpf) exposed for 1 h at 28 °C through direct administration routes via microinjection into the caudal vein (**a**), heart ventricle (**b**), and hindbrain (**c**). The presented sections originate from one representative larva per condition. The images were generated by preparing a colormap from blue (no detection) to yellow (high local concentration), and images were further processed in 96 dpi resolution with 24 bit color under no denoising state. Each panel shows two different slices of the same specimen.

**Figure 6:**
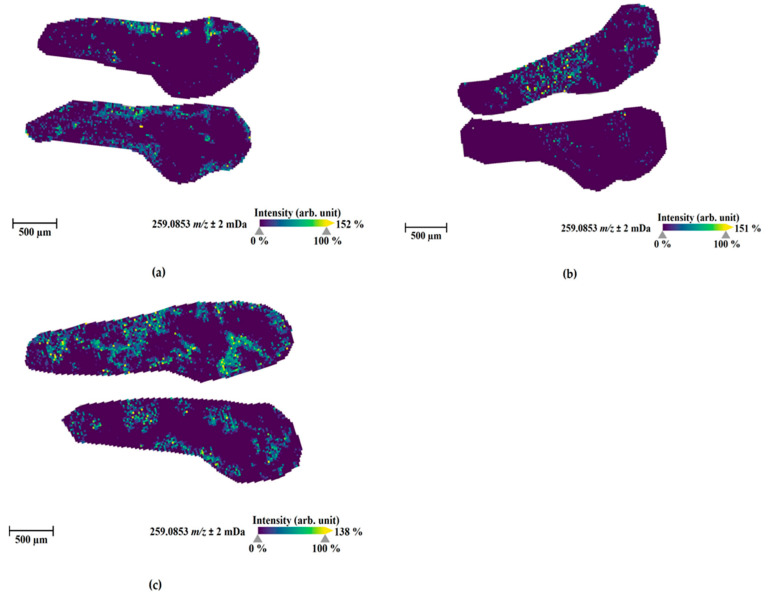
MALDI-MS images of the most abundant metabolite M1 (amide hydrolysis form, sodium adduct, *m/z* 259.0853) in zebrafish larvae at 4 days post-fertilization (dpf) exposed by 4F-MDMB-BINACA for 1 h at 28 °C through direct administration routes via microinjection into the caudal vein (**a**), heart ventricle (**b**), and hindbrain (**c**). The presented sections originated from one representative larva per condition. The images were generated by preparing a colormap from blue (no detection) to yellow (high local concentration), and images were further processed in 96 dpi resolution with 24 bit color under no denoising state. Each panel shows two different slices of the same specimen.

One hour after microinjections into three internal organs, the parent compound (Figure 5) and three metabolites (M1 (amide hydrolysis from M3; Figure 6), M3 (Supplementary Figure S2), and M4 (Supplementary Figure S3)) could be visualized in the sections from these injected ZF larvae. M1 was uniquely detected in the injected ZF larvae prepared for MALDI-MSI among all samples. Moreover, M3 and M4 were observed in all injected ZF larvae analyzed by LC-HRMS/MS and MALDI-MSI and these metabolites were also found in human urine samples and blood samples, respectively (Table 1). However, corresponding masses of M12, M15, and M20 as the major metabolites detected in LC-HRMS/MS were not found by MALDI-MSI.

The parent compound, 4F-MDMB-BINACA, was mainly located at the ZF larva’s peripheral region (Figure 5). In contrast, the most abundant metabolite in MSI, M1, was detected throughout the larval body, and it was distributed in all compartments spreading from the head to the tail region. ZF larva injected into the hindbrain showed the most prominent spread of M1, and in sections from ZF larvae injected into the heart ventricle, M1 somehow accumulated more in the dorsal section than in other sections (Figure 6). M3 and M4 were evenly distributed in the ZF larval bodies, showing some accumulation in the head region with relatively minor intensity (Supplementary Figures S2 and S3). Overall, hindbrain injection resulted in the best distribution of these metabolites, which is in line with the findings from LC-HRMS/MS measurements (Figure 3). The widely distributional detection of these metabolites inside the larvae, despite the local and low abundance detection of 4F-MDMB-BINACA, leads to the assumption that the circulation of the parent compound proceeded sufficiently within 1 h after microinjection, allowing for fast and efficient metabolism.

When we compared the detection of metabolites from MALDI-MSI experiments and LC-HRMS/MS studies, we observed a discrepancy with respect to the main ion adduct formation of the detected metabolites. Furthermore, different metabolites were detected as most abundant, and this dissimilarity could be explained by the use of entirely different workup steps and analytical methodologies, e.g., sample preparation and separation, ionization techniques, and data collecting/processing, just to name a few.

Common adducts in MSI spectra are formed with protons, sodium, and calcium derived from inorganic salts or residual water, naturally present in all biological samples. Reducing the formation of inorganic salt adducts in MSI is therefore a crucial step for enhancing sensitivity [47,48]. For MALDI ionization preferably leading to [M+H]^+^ ion species, a matrix deposition step is required. Here, we used 2,5-dihydroxybenzoic acid (DHB) [28], which is widely used, especially for metabolites and peptides in positive ionization mode [45,47,48]. As expected, in previous MALDI-MSI measurements of 7′*N*-5F-ADB, we mainly found protonated adducts of its metabolites, including the parent compound [28]. In contrast, MSI with 4F-MDMB-BINACA resulted in [M+Na]^+^ as predominant ion species; however, [M+H]^+^ ions were detected with high abundance. Thus, 4F-MDMB-BINACA and its metabolites were screened by MALDI-MSI, based on masses of their respective sodium adduct ions generated from structures identified by LC-HRMS/MS/MS^2^ [27] (Supplementary Table S2). In future studies, other matrices such as sinapinic acid (SA), α-cyano-4-hydroxycinnamic acid (CHCA), and commercial matrix mixtures (e.g., DHB:CHCA = 1:1) could be considered to improve the detection of 4F-MDMB-BINACA in ZF samples. Another reason that MSI detection of metabolites was less successful than analyses based on LC-HRMS/MS could be the different sample size; for MSI, one ZF larva was sectioned into on average ten slices, whereas 30 ZF larvae were pooled for LC-HRMS/MS measurements. Further studies will be needed to optimize MSI for the assessment of ZF larvae considering various classes of drugs. We plan to further investigate MSI for ZF larvae by applying existing and optimized methods using ZF larvae homogenates [49,50,51] in order to account for possible deviations based on different tissue types [49] and local interfering molecules [45].

### 2.3. Differences in the Metabolism of 4F-MDMB-BINACA and 7′N-5F-ADB in Zebrafish Larvae and Influence of Cannabinoid Receptor Function on Drug Metabolism

While investigating the ZF larvae model employing various administration routes, we confirmed the highest number of metabolites of 7′*N*-5F-ADB, 4F-MDMB-BINACA, and other NPSs in the ZF larvae compared to other models [25,26,27,28]. The overall number of detected metabolites of 4F-MDMB-BINACA was lower than for 7′*N*-5F-ADB (Supplementary Table S3), despite their high structural similarity. Moreover, the calculated physicochemical properties of both SCs (Table 2) are similar. In particular, they have a similar predicted log *p* value (lipophilicity), which possibly could have influenced drug distribution and metabolism in the ZF larvae model. In detail, in human urine samples and the microinjected ZF larvae, 27 and 24 metabolites out of 36 possible metabolites for 7′*N*-5F-ADB, and nine and 11 out of 26 possible metabolites for 4F-MDMB-BINACA were observed, respectively.

**Figure 7:**
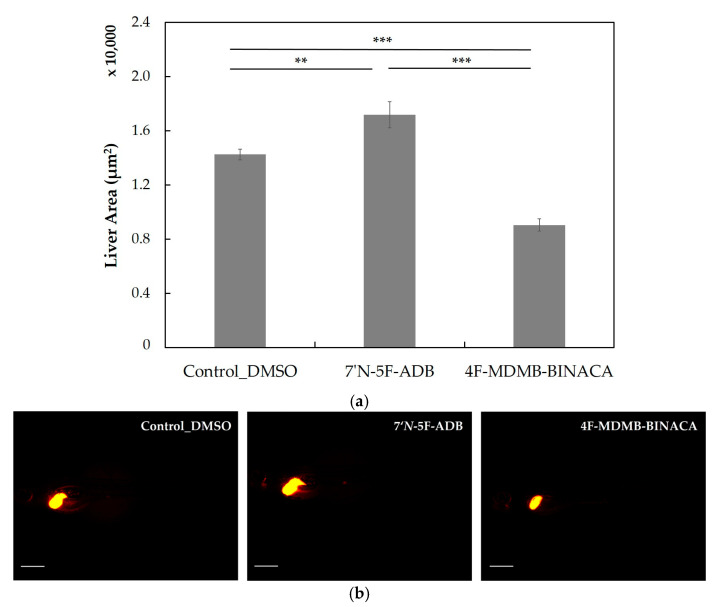
Liver size assessment (**a**) and red fluorescent liver morphologies (**b**) of transgenic ZF larvae (Tg(*fabp10a*:DsRed;*elaA*:EGFP)) treated by waterborne exposure with DMSO, 7′*N*-5F-ADB (50 µM), or 4F-MDMB-BINACA (25 µM) from 4 dpf to 5 dpf (n = 11–15). All data are represented by the mean ± standard error of the mean (s.e.m.) and *p* values were computed by one-way ANOVA (** *p* < 0.01, *** *p* < 0.001). Liver sizes were determined based on fluorescence microscopy images using ImageJ 1.53a. Scale bars: 500 µm.

Due to the relatively small number of detected 4F-MDMB-BINACA metabolites in the ZF larvae model, we investigated a possible correlation between (impaired) liver development and metabolic function in ZF larvae. Thus, we evaluated the SCs hepatotoxic effect along with 7′*N*-5F-ADB. The ZF liver toxicity of these two SCs was determined indirectly by measuring the liver size after treatment via conventional waterborne exposure using a transgenic ZF line (Tg(*fabp10a*:DsRed;*elaA*:EGFP)) (Figure 7). This transgenic line displays strong constitutive expression of red fluorescent protein (DsRed) in the liver and enhanced green fluorescent protein (EGFP) in the exocrine pancreas under the *fabp10a* promoter and the *elaA* promoter, respectively, and it is commonly used for fluorescence-based in vivo hepatotoxicity assays [52,53,54]. In these ZF larvae treated for 1 d with 7′*N*-5F-ADB, the liver size increased by 21%, whereas larvae treated with 4F-MDMB-BINACA displayed a 37% decrease in liver size. These findings in ZF larvae treated with 4F-MDMB-BINACA resembled a phenotype that was previously described by Liu et al., which was caused by the loss of two cannabinoid receptors (CB1 and CB2) in ZF larvae [38]. Despite a reduction of liver size, the authors also reported impaired hepatocyte differentiation and proliferation in these larvae, whereas the abnormal liver architecture influenced the development of ZF even up to the adult stage. Based on the acute effect of 4F-MDMB-BINACA on liver development in 4 dpf larvae, we thought that this phenotype might explain the less efficient drug metabolism compared to 7′*N*-5F-ADB. Although treatment with the latter also induced a phenotype in ZF larvae the extent of the effect was less pronounced than observed in ZF larvae treated with 4F-MDMB-BINACA. The generally contrary effects of both SCs that serve as CB receptor agonists, with 4F-MDMB-BINACA reducing liver size and 7′*N*-5F-ADB increasing it cannot be currently explained. In principle, it was expected that 4F-MDMB-BINACA, due to its agonistic CB receptor activity, also increases the liver size in ZF larvae. However, complex regulation following activation of CB1 and/or CB2 and the balance between activity of both receptors might explain the differential phenotypes observed. For future studies, it would be interesting to study the influence of CB receptor antagonists reducing the liver size on ZF metabolism and to compare the results to our findings with 4F-MDMB-BINACA, for which we found an atypical phenotype.

In order to study the possible impact of CB1/2 receptor function on drug metabolism in more detail, we employed the morpholino (MO) technology to knock down *cnr1* and *cnr2* in ZF larvae. For determining the optimal non-toxic conditions for the gene knockdown, the survival rates of ZF larvae from 3 dpf to 4 dpf were examined after early-stage injection of three different concentrations of MOs (Supplementary Figure S4). Simultaneously, morphological malformations of larvae were recorded (Supplementary Table S4). CB2-deficient morphants showed morphological malformations, such as curved or vent spine, shortened tail, and edemas in the pericardial and yolk sac more frequently than the CB1-deficient morphants (Supplementary Figure S5). Considering these toxic effects of high MO dosage, we used an MO concentration of 100 µM for all subsequent experiments. Furthermore, since the random MO control 25-*N* still indicated some rare toxic effects on ZF larvae, control injections were performed with distilled water unless otherwise specified in the Supplementary Table S5.

**Figure 8:**
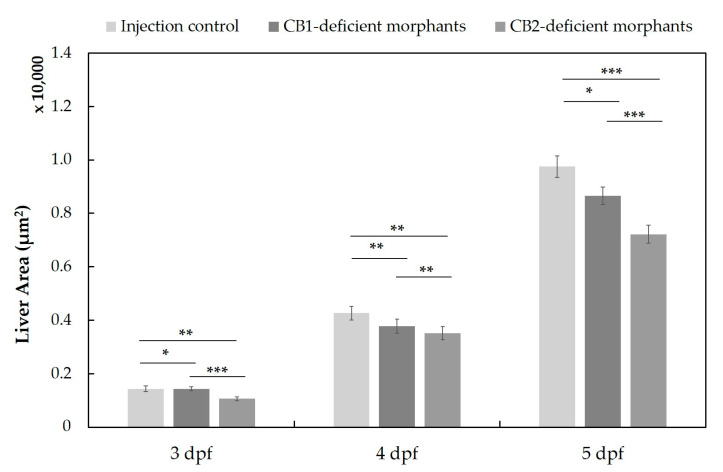
Liver size assessment in the transgenic ZF line (Tg(*fabp10a*:DsRed;*elaA*:EGFP)) after MO injection at one-cell stage to generate CB1- and CB2-deficient morphants (n = 43 (at 3 dpf), 41 (at 4 dpf), 55 (at 5 dpf)). All data are represented by the mean ± standard error of the mean (s.e.m.), and *p* values were computed with one-way ANOVA (* *p* < 0.05, ** *p* < 0.01, *** *p* < 0.001). Liver sizes were determined based on fluorescence microscopy images using ImageJ 1.53a.

Following *cnr1* and *cnr2* gene knockdown in embryos of the Tg(*fabp10a*:DsRed; *elaA*:EGFP) ZF line, we assessed the liver size of morphants from 3 to 5 dpf (Figure 8). We observed small effects on the liver size already at 3 and 4 dpf, whereas the impact of CB1 and CB2 deficiency was most obvious at 5 dpf, and CB2-deficient morphants showed a significantly reduced liver area compared to the control group (mock injection with distilled water). The extent of liver size reduction in 5 dpf CB2-deficient morphants (26% reduction compared to control) was similar to that observed in 5 dpf larvae treated for 1 d with 4F-MDMB-BINACA (37% reduction compared to untreated control).

**Figure 9:**
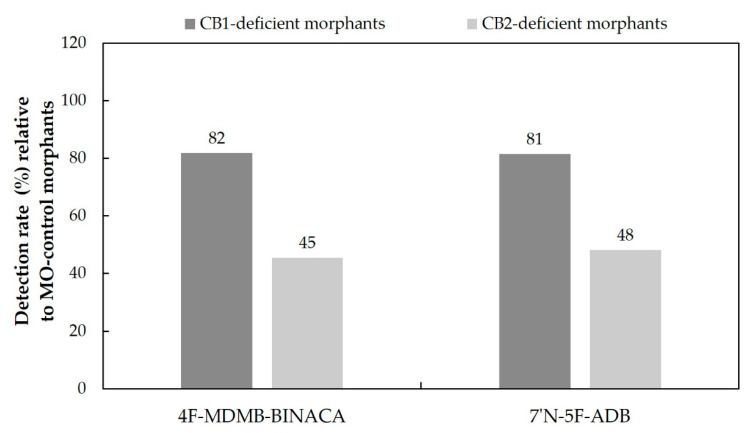
Comparison of the overall detection rates of phase I and phase II metabolites produced in CB1-deficient and CB2-deficient ZF larvae after waterborne exposure to 25 µM 4F-MDMB-BINACA and 50 µM 7′*N*-5F-ADB, respectively (n = 3). The detection rates were calculated based on the number of metabolites found in the respective control population (set to 100%).

To demonstrate the effect of CB1- and CB2-deficiency on SC metabolism, we exposed both morphants to 4F-MDMB-BINACA and 7′*N*-5F-ADB, respectively, from 4 to 5 dpf as described in previous studies, and we determined the metabolite profile by LC-HRMS/MS [25,27]. The direct administration route via injection was not considered in this study to avoid the stress of an additional injection. Remarkably, the influence of these two cannabinoid receptors on the overall number of detected metabolites of 4F-MDMB-BINACA and 7′*N*-5F-ADB compared to a control group (mock-injected MO control) was pronounced. We observed only ca. 80% and ca. 45% of metabolites of both SCs in the CB1-deficient and CB2-deficient morphants, respectively (Figure 9 and Supplementary Figure S6). SCs are generally described to mainly act as agonists of CB1 and as partial agonists of CB2 [30,55]. However, in the given example (Figure 9), dysfunctional CB2 seems to play a more critical role in liver metabolic efficiency of ZF larvae than CB1. As NPSs exert their psychoactive effects mostly via CB1, their activity on CB1 function was mainly investigated using in vitro assays [30,55,56]. Cannaert et al. reported 4F-MDMB-BINACA as one of the most potent CB1 agonists in recently detected twelve SCs with a half-maximal effective concentration (EC_50_) of 7.37 nM, compared to 5475 nM for 7′*N*-5F-ADB [55]. In this respect, it seems plausible that 4F-MDMB-BINACA might also serve as a stronger CB2 agonist in ZF larvae than 7′*N*-5F-ADB, and the strong activation of CB2 might have a similar effect on drug metabolism as CB2 deficiency, both leading to a hepatotoxic phenotype.

## 3. Materials and Methods

### 3.1. Chemicals and Other Materials

4F-MDMB-BINACA was provided by the EU-funded project ADEBAR (IZ25-5793-2016-27) for research purposes. 7′*N*-5F-ADB was obtained from www.buyreserchchemicals.de (accessed on 19 January 2022) tagged as 4′*N*-5F-ADB, which was confirmed to be 7′*N*-5F-ADB by analysis of NMR (nuclear magnetic resonance) [25]. Dimethyl sulfoxide (DMSO), methylene blue, phenol red, tricaine (3-amino-benzoic acid ethyl ester), mineral oil, trifluoroacetic acid, gelatin from cold water fish skin, and 2,5-dihydroxybenzoic acid (2,5-DHB) were obtained from Sigma-Aldrich (Taufkirchen, Germany). Methanol (LC-MS grade), acetonitrile (LC-MS grade), formic acid (LC-MS grade) were from VWR (Darmstadt, Germany). NaCl, KCl, MgSO_4_, Ca(NO_3_)_2_, and HEPES were obtained from Carl Roth (Karlsruhe, Germany). The 10 mM stock solutions for all standards were prepared in DMSO, and solutions were stored for a maximum of one month at −20 °C. The working solutions were freshly prepared prior to each experiment. Morpholino oligonucleotides (MOs) were purchased from Gene Tools, LLC (Philomath, OR, USA). The working solution of MOs was prepared at 1 mM in distilled water and stored according to the manufacturer’s protocol, and the detailed information of MOs used in the present study is given in Section 3.5. 6-well plates were obtained from Sarstedt (Nümbrecht, Germany). Glass capillaries TW100F-4 (4 inches (100 mm), 1/0.75 OD/ID (mm), Filament) were from World Precision Instruments Germany GmbH (Friedberg, Germany). Conductive indium-tin-oxide (ITO) coated glass slides were purchased from Bruker Daltonics (Bremen, Germany). Zebrafish embryos of the AB wild-type line were initially obtained from the Luxembourg Center for Systems Biomedicine (Belvaux, Luxembourg). The ZF line Tg(*fabp10a*:DsRed;*elaA*:EGFP) was provided by the Goessling lab (Boston, MA, USA). Dry small granulate food was purchased from SDS Deutschland (Limburgerhof, Germany), and *Artemia* cysts (>230,000 nauplii per gram) were obtained from Coralsands (Wiesbaden, Germany).

### 3.2. Zebrafish Maintenance and Embryo Collection

Zebrafish husbandry and all experiments with ZF larvae were performed according to EU Directive 2010/63/EU and the German Animal Welfare Act (§11 Abs. 1 TierSchG). All works were accomplished following internal standard-operating procedures (SOPs) based on published standard methods [57]. Adult ZF were kept in an automated aquatic eco-system (PENTAIR, Apopka, UK) that is continuously monitored: Temperature (27 ± 0.5 °C), pH (7.0 ± 0.1), conductivity (800 ± 50 µS), and light–dark cycle (14 h/10 h). Fish were fed twice a day with dry small granulate food and freshly hatched live *Artemia* cysts once a day. The ZF embryo/larvae medium (0.3× Danieau’s solution) was composed of 17 mM NaCl, 2 mM KCl, 0.12 mM MgSO_4_, 1.8 mM Ca(NO_3_)_2_, 1.5 mM HEPES, pH 7.1–7.3, and 1.2 µM methylene blue. For ZF embryo production, ZF pairs were kept overnight in standard mating cages, separated by gender. The following morning, the adult ZF started spawning immediately after removing the separators. All fertilized eggs of ZF were sorted using a Zeiss Stemi 508 stereo microscope (Carl Zeiss Microscopy GmbH, Jena, Germany). All embryos were raised in an incubator at 28 °C with daily medium change to clean embryo cultures. ZF larvae at 4 dpf were used for drug metabolism studies.

### 3.3. Drug Treatment of Zebrafish Larvae via Medium Exposure

The sample preparation following aquatic drug exposure is described elsewhere [25,27,28]. A non-toxic exposure concentration was chosen based on the survival rate as determined by in vivo maximum-tolerated concentration (MTC) experiments with 4 dpf ZF larvae. For the two drugs (4F-MDMB-BINACA, 7′*N*-5F-ADB) investigated in the current study, the results of MTC are published in our previous papers [25,27]. For metabolite studies, 15 ZF larvae at 4 dpf were transferred to one well of a 6-well plate containing 3 mL of 0.3× Danieau’s medium with 25 µM 4F-MDMB-BINACA and 50 µM 7′*N*-5F-ADB, respectively. All exposure media contained a final concentration of 1% (*v*/*v*) DMSO, and ZF larvae were treated for 24 h in an incubator at 28 °C. An additional 15 larvae were incubated in a compound-free medium containing only 1% DMSO (*v*/*v*) as negative control (background masses). Before sample extraction, 30 larvae were pooled, and sample extractions were performed as described below in Section 3.7. All pooled samples were prepared in triplicates.

### 3.4. Drug Treatment of Zebrafish Larvae via Microinjections into Different Compartments

For microinjections, the glass microneedle was produced by a Flaming/Brown type micropipette puller (Model P-100, Sutter Instrument, Novato, CA, USA). The microneedle can be fabricated to fit the purpose via changes of heating and pulling parameters. 4F-MDMB-BINACA solution at 5 mM was prepared in 50% DMSO and 50% of a 0.5% phenol red solution, and the microneedle was filled with this solution without air bubbles by a microloader pipette. The needle containing the solution was assembled in an M-152 manipulator (Narishige Group, Tokyo, Japan) connected to a FemtoJet 4× Microinjector (Eppendorf, Hamburg, Germany). Before starting injections, every microinjection needle was calibrated by a single droplet injection onto mineral oil on a micrometer slide. We chose to microinject 4.19 nL of 5 mM 4F-MDMB-BINACA per one larva, which corresponds to a total amount of 228.4 ng in a pool of 30 larvae. The detailed conditions of making the glass microneedle and needle calibration are given elsewhere [28].

Before starting the injection, ZF larvae at 4 dpf were anaesthetized by tricaine solution, and promptly, they were aligned on an agarose plate prepared using Z-MOLD (World Precision Instruments, Sarasota, FL, USA). The excess medium was removed with a pipette as much as possible to immobilize ZF larva during the injection. Microinjections were conducted into three different compartments of ZF larvae (caudal vein, heart ventricle, and hindbrain) under a stereo microscope (Zeiss Stemi 508 stereo microscope). The injected larvae were immediately transferred to fresh 0.3× Danieau’s medium, and they were incubated at 28 °C for 1 h. Prior to sample extraction, all malformed larvae generated after the injection were excluded by sorting using a microscope, and 30 healthy larvae were pooled into one tube. Sample extractions proceeded as described below (Section 3.7), and all pooled larvae were prepared in triplicates. In general, the mortality rate of ZF larvae after microinjection was below 10% in all cases.

### 3.5. Gene Knockdown of Cannabinoid Receptor Type 1 and Type 2 via Microinjection of Morpholino Oligonucleotides in Zebrafish Embryos

The morpholino oligonucleotides (MOs; synthesized by Gene Tools) against cannabinoid receptor type 1 (CB1) and type 2 (CB2) were designed to target its splicing site (Supplementary Table S5), and these MOs were validated in earlier studies [38,58]. Each MO working solution, yielding final concentrations of 100, 200, and 500 µM, was freshly prepared from a 1 mM stock solution according to the manufacturer’s recommendations and a previously published protocol [59] and mixed with 0.5% phenol red solution as described in Section 3.4. These MO solutions were injected into one-cell stage embryos with a volume of 4.19 nL. Embryos were maintained as described above (Section 3.2).

### 3.6. Measurement of the Fluorescent Liver Size in a Transgenic Zebrafish Larva

A transgenic zebrafish line Tg(*fabp10a*:DsRed*; elaA*:EGFP) was used to determine the liver size of ZF larva, which has been widely employed for liver studies [52,53,54]. This ZF line expresses the red fluorescent protein (DsRed) under control of the *fabp10a* promoter in the liver and the enhanced green protein (EGFP) under the elastase A (*elaA*) promoter in the exocrine pancreas. For the fluorescent liver measurement of ZF larvae from 3 dpf to 5 dpf, ZF larvae were anesthetized on ice. All ZF larvae were arranged directly in a manner to minimize measuring variances, in which the head part was positioned on the left side and the end tail on the right side. Images of ZF livers were taken using a Leica M205 FA stereo microscope (Leica Mikrosysteme Vertrieb GmbH, Wetzlar, Germany) connected with an X-Cite^®^ 200DC illuminator (Excelitas technologies, Mississauga, ON, Canada) and appropriate filter sets for red fluorescent protein detection. The liver size of ZF larva was then quantified using ImageJ version 1.53a [60].

### 3.7. Zebrafish Sample Preparation and Metabolite Analysis by LC-HRMS/MS

All treated larvae (waterborne exposure and microinjection) were transferred into a tube using a pipette and then rinsed twice with 1 mL of 0.3× Danieau’s solution. The washed larvae were euthanized by placing the tubes in ice water for 15 min. These larvae were snap-frozen in liquid nitrogen, followed by lyophilization for 4 h. The lyophilized larvae were stored for a maximum of one week at −20 °C before extraction. For metabolite identification, frozen larvae were thawed at room temperature for at least 30 min and extracted by vigorous vortexing for 2 min with 50 µL methanol. The sample was centrifuged at 10,000× *g* for 2 min at room temperature, and the supernatant was transferred to an autosampler vial. The extract from ZF larvae was kept in a freezer at −20 °C and analyzed within one week after extraction.

The LC-HRMS/MS system consisted of a Dionex Ultimate 3000 RSLC system (Thermo Fisher Scientific, Germering, Germany) and maXis 4G HR-QTOF mass spectrometer (Bremen, Germany) with the Apollo II ESI source. Separation of 5 µL aliquot from all larvae extracts was carried out by a linear gradient with 0.1% formic acid in water (*v*/*v*, eluent A) and 0.1% formic acid in acetonitrile (*v*/*v*, eluent B) at a flow rate of 600 µL/min. As stationary phase, a Waters ACQUITY BEH C_18_ column (100 × 2.1 mm, 1.7 µm) equipped with a Waters VanGuard BEH C_18_ 1.7 µm guard column at 45 °C was used. The gradient mode was programmed into these steps as follows; 0–0.5 min, 5% eluent B; 0.5–18.5 min, 5–95% eluent B; 18.5–20.5 min, 95% eluent B; 20.5–21 min, 95–5% eluent B; 21–22.5 min, 5% eluent B. UV spectra were recorded by a Diode array detector (DAD) in the range from 200 to 600 nm. Mass spectra were recorded in centroid mode ranging from 150–2500 *m*/*z* at a 2 Hz full scan acquisition rate under auto MS/MS conditions in positive ionization mode. External calibration was automatically performed by injecting sodium formate and calibration on the respective clusters formed in the ESI source before every LC-HRMS/MS run. All MS analyses were acquired in the presence of the *m*/*z* 622.0290, 922.0098, and 1221.9906 ions as the lock masses generated with the [M+H]^+^ ions of C_12_H_19_F_12_N_3_O_6_P_3_, C_18_H_19_O_6_N_3_P_3_F_2_, and C_24_H_19_F_36_N_3_O_6_P_3_. DataAnalysis software version 4.4 (Bruker Daltonics, Bremen, Germany) was used for qualitative analysis. All data were presented with the range of mean and standard deviation (SD) using MS Excel 2016.

All masses and MS^2^ data of four substrates, 4F-MDMB-BINACA, 7′*N*-5F-ADB, and its expected metabolites, were confirmed in our previous studies [25,27,28,61]. Accordingly, their structures and other detailed information were not included in the present study. With the absence of reference standards for using absolute quantification, the comparisons of the metabolites performed in this paper were executed by quantifying peak areas under the assumption of similar ionization behaviors of the individual metabolites.

### 3.8. Mass Spectrometry Image Analysis of Zebrafish Larva by MALDI-FT-ICR

The ZF larvae treated by the procedures described in Section 3.3 and Section 3.4 were embedded in 40% (*w*/*v*) gelatin solution and were then frozen and stored at −20 °C until cryosectioning. A single larva was cut with 10-µm thickness at −20 °C using a cryostat (MEV; SLEE, Mainz, Germany), and every section was placed on a cold conductive indium-tin-oxide (ITO) coated glass slide. The slides were scanned under a microscope for alignment of the optical image of the sample in MALDI. The serial sections from one larva in one glass slide were deposited using TM-Sprayer (HTX M5; HTX Technologies, Chapel Hill, NC, USA) with 15 mg mL^−1^ 2,5-dihydroxybenzoic acid (2,5-DHB) in acetonitrile:water (9:1, *v*/*v*) solution containing 0.1% of trifluoroacetic acid, and then dried in a vacuum desiccator for ≥2 h. The dried glass slide was stored at −20 °C before MALDI measurement. Importantly, as the detachment of the sections from the glass slide happened during the thawing step prior to the MALDI analysis, it is recommended to analyze the samples within one week after matrix deposition. This phenomenon occurred mainly from the arid part of the section embedded in a 40% gelatin medium.

For MSI measurement, the sections of ZF larvae were analyzed by MALDI and 7T SolariX FT-ICR (Bruker Daltonics, Bremen, Germany) in positive ionization mode (*m/z* range 150–1000), using 40 laser shots per pixel with a raster width of 20 µm. Before acquiring MALDI images, the mass calibration of FT-ICR was carried out using the calibration standard according to the manufacturer manual, and for auto-calibration of MALDI of each laser measurement, the lock mass was set to *m/z* 273.0394 (2,5-DHB matrix). ftmsControl version. 2.2.0, flexImaging version 5.0, and SCiLS Lab version 2021a Pro software (Bruker Daltonics, Bremen, Germany) were used for MALDI-MSI data acquisitions and image data analyses in two dimensions.

## 4. Summary and Conclusions

Our study aimed to strengthen the understanding of the human metabolism of 4F-MDMB-BINACA using the ZF larvae model as an alternative in vivo model. By predicting human metabolism based on results in the ZF larvae model, several issues caused by variance in human biosamples due to the lack of homogeneity and diversity among samples in random sampling could be overcome. ZF larvae were treated with the SC by direct injection into vital organs, and in total, ten metabolites were observed (Figure 2). Encouragingly, we found high matching rates of ZF metabolites of 67% for human blood samples and 56% for human urine samples, respectively. In conclusion, metabolite data sets from the ZF model, in combination with data from in vitro models such as HepaRG, can complement and extend human metabolism data.

Moreover, the spatial distribution of 4F-MDMB-BINACA and its metabolites in ZF larvae was analyzed by MALDI-MSI, and three metabolites were visualized inside ZF larval bodies by MSI (Figure 6 and Supplementary Figures S2 and S3). In the context of our study, this technology can be applied to analyze the biodistribution of drugs and their metabolites and it can help to investigate, e.g., accumulation of compounds in specific organs. Here, we did not detect unusual local concentrations of the studied SC, but we were able to identify another metabolite (M1), which was not found in LC-HRMS-based screening of extracted larvae.

The overall metabolite detection between 4F-MDMB-BINACA and another SC, 7′*N*-5F-ADB [28], were contrasting despite the similarity of their physicochemical properties (Table 2). Consequently, we performed additional experiments to shed light on the observed differences in ZF metabolism with respect to the total number of detected metabolites for these two SCs. The liver toxicity of the SCs was determined indirectly by measuring the liver size of a transgenic ZF line, and 4F-MDMB-BINACA induced a pronounced liver size reduction despite its strong agonistic activity on CB2 (Figure 7). Thus, we investigated the role of cannabinoid receptors on liver development and drug metabolism in ZF larvae. We could show that CB2-deficient larvae showed a drastic decline in the overall number of detected metabolites of both SCs, which could be related to the simultaneously observed hepatotoxic effect (Figure 9 and Supplementary Figure S6). The role of CB2 deficiency (and dysregulation) in drug metabolism requires further research, and here, we provide first evidence that it might play a major role in the overall efficacy of drug metabolism in the liver.

In summary, we applied established methodologies for studying drug metabolism in ZF larvae. A concise evaluation in wild-type ZF allowed for refinement of the phase I metabolic pathways of 4F-MDMB-BINACA. Additionally, we investigated the role of cannabinoid receptor function in metabolism using genetically modified ZF larvae. We gained important insights into the relationship between liver function, cannabinoid receptor function, and the onset of drug metabolism. Although many questions associated with various biological systems for drug metabolism studies require further analyses, this study emphasizes the high potential of ZF larvae as a model to study complex human bioprocesses. We expect that ZF will play a more prominent role in translational research in future. Importantly, applying state-the-art analytics contributes to a better understanding of drug pharmaco- and toxicokinetics, which, in turn, is necessary to understand complex biological processes in the in vivo model.

## Figures and Tables

**Table 1:** Summary of 4F-MDMB-BINACA and its phase I and II metabolites and their detection in human biosamples and microinjected zebrafish larvae.

Compounds	Metabolite ID	Integrated Human Screening Data * [27,32,34,35]		Data from Zebrafish Larvae			
				Aquatic Exposure, Published Data [27]	Microinjection		
		Blood	Urine		Caudal Vein	Heart Ventricle	Hindbrain
Parent compound	4F-MDMB- BINACA			+++	+++	+++	+++
Phase I metabolites	M1						
	M2			+	+ ^nq^		+ ^nq^
	M3		√√		+ ^nq^	+ ^nq^	+ ^nq^
	M4	√√		+	+	+	+
	M5			+			
	M6	√	√√	++	+	+	+
	M7		√	+			
	M8	√	√	+			
	M9			+			
	M10		√√	+	+ ^nq^	+ ^nq^	+ ^nq^
	M11		√√	+	+	+	+
	M12			+	+	+	+
	M13		√	+			
	M14		√				
	M15		√√	+	+	++	+
	M16			+			
	M17						
	M18			+			
	M19						
Total number of phase I metabolites		3	9	14	8	7	8
Phase II metabolites	M20			+	++	+	+
	M21						
	M22						
	M23						
	M24			+			
	M25			+	+ ^nq^	+	+
	M26			+			
Total number of phase II metabolites		-	-	4	2	2	2
Total number of detected Phase I/II metabolites		3	9	18	10	9	10

* All human data were taken from recently published studies; integrated blood data were quoted from [27,32], integrated urine data from [27,32,34,35], and details are shown in Supplementary Table S1. √: detected at least twice among the samples in each respective human matrix; √√: detected in both the microinjected ZF larvae and human biosamples; ^nq^: confirmed mass, but not quantified due to peak detection below signal-to-noise ratio of 3. +: Peak detected, ++: second most abundant peak among metabolites, +++: most abundant peak among metabolites.

**Table 2:** Comparison of calculated physicochemical properties ^1^ of 4F-MDMB-BINACA and 7′*N*-5F-ADB.

	4F-MDMB-BINACA	7′*N*-5F-ADB
Log *P* ^2^	3.12	3.20
Strongest acidic pKa ^3^	14.65	15.05
Strongest basic pKa ^3^	−0.76	3.11
Log Intrinsic solubility (mol/L) ^4^	−4.37	−4.50

^1^ All data were calculated online at https://chemicalize.com (accessed on 31 October 2019). ^2^ Log *P* value refers to the logarithm of the partition coefficient of a compound in octanol and water. ^3^ pKa, the acid dissociation constant at logarithmic scale, refers to a specific equilibrium constant to represent the ability of an acid to dissociate protons from a heterocyclic core (acidic pKa) and the protonated tertiary base (basic pKa). ^4^ Intrinsic solubility is the basic solubility of a drug when it is entirely unionized.

## Data Availability

Not applicable.

## Supplementary Materials

The following are available online, Figure S1: Detection profile of three minor metabolites of 4F-MDMB-BINACA in microinjected zebrafish larvae, Figure S2: MALDI-MS images of the most abundant metabolites M3 (ester hydrolysis in combination with *N*-dealkylation, sodium adduct, *m/z* 298.1162) in zebrafish larvae at 4 days post-fertilization (dpf), Figure S3: MALDI-MS images of the most abundant metabolites M4 (*N*-dealkylation form, sodium adduct, *m/z* 312.1319) in zebrafish larvae at 4 days post-fertilization (dpf), Figure S4: The survival rates of zebrafish mutant larvae at 4 days post-fertilization (dpf) according to the different concentrations of each morpholino oligonucleotide, Figure S5: Exemplarily morphological malformation images of zebrafish mutant larvae microinjected by morpholino oligonucleotides (MOs) at 5 days post-fertilization (dpf), Figure S6: Mutual comparability of the overall metabolites detected in CB1-deficient and CB2-deficient ZF larvae after waterborne exposure of 4F-MDMB-BINACA (at 25 µM) and 7′*N*-5F-ADB (at 50 µM) using Venn diagrams, Table S1: The detailed information of 4F-MDMB-BINACA and its phase I and phase II metabolites and their detection in human biosamples published in literature, Table S2: Mass list of 4F-MDMB-BINACA and its phase I and phase II metabolites used for LC-HRMS/MS and MALDI-FT-ICR measurements, Table S3: Comparison of the total number of metabolites detected in zebrafish larvae and human urine samples for 7′*N*-5F-ADB and 4F-MDMB-BINACA, Table S4: Comparison of the morphological malformation cases in the zebrafish mutant larvae from 3 days post-fertilization (dpf) to 4 dpf after MOs injection at a one-cell stage, Table S5: Sequences of morpholino oligonucleotides (MOs) for the gene knockdown of CB1 and CB2 by binding its splicing site.

## Sample Availability

The two compounds, 4F-MDMB-BINACA and 7′*N*-5F-ADB, are available through commercial sources.
