# Supplementary material for: Induction of Liver Size Reduction in Zebrafish Larvae by the Emerging Synthetic Cannabinoid 4F-MDMB-BINACA and Its Impact on Drug Metabolism
Source: Molecules. 2022 Feb 15;27(4):1290. doi: 10.3390/molecules27041290 (PMC8879502; doi:10.3390/molecules27041290)
Supplement: Supplementary file 1 [file molecules-27-01290-s001.zip › molecules-1583585-supplementary.pdf]

molecules

MDPI

Supplementary Material

Induction of liver size reduction in zebrafish larvae by the emerging synthetic cannabinoid 4F-MDMB-BINACA and its impact on drug metabolism

Yu Mi Park [1,2,3], Charlotte Dahlem [4], Markus R. Meyer [5], Alexandra K. Kiemer [4], Rolf Müller [1,3,6,*] and Jennifer Herrmann [1,6,*]

[1] Helmholtz Institute for Pharmaceutical Research Saarland (HIPS), Helmholtz Centre for Infection Research, Saarland University Campus, 66123 Saarbrücken, Germany; Yu-Mi.Park@helmholtz-hips.de
[2] Environmental Safety Group, Korea Institute of Science and Technology (KIST) Europe, 66123 Saarbrücken, Germany
[3] Department of Pharmacy, Saarland University, 66123 Saarbrücken, Germany
[4] Department of Pharmacy, Pharmaceutical Biology, Saarland University, Campus C2 3, 66123 Saarbrücken, Germany; charlotte.dahlem@uni-saarland.de; pharm.bio.kiemer@mx.uni-saarland.de
[5] Department of Experimental and Clinical Toxicology, Institute of Experimental and Clinical Pharmacology and Toxicology, Center for Molecular Signaling (PZMS), Saarland University, 66421 Homburg, Germany; m.r.meyer@mx.uni-saarland.de
[6] German Center for Infection Research (DZIF), 38124 Braunschweig, Germany
* Correspondence: Rolf.Mueller@helmholtz-hips.de (R.M.); Jennifer.Herrmann@helmholtz-hips.de (J.H.)

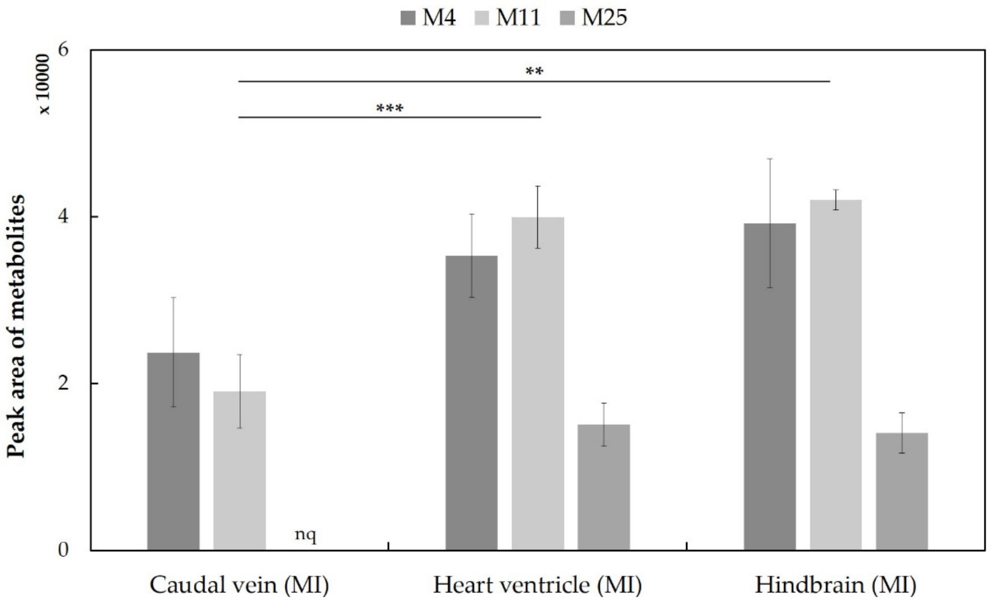

Figure S1. Detection profiles of the three minor abundant metabolites of 4F-MDMB-BINACA in microinjected zebrafish larvae (caudal vein, heart ventricle, and hindbrain). These three minor metabolites, M4, M11, and M25, were formed by *N*-dealkylation, oxidative defluorination, hydroxylation of the tertiary butyl part in combination with glucuronidation isomer 1, respectively. The clustered columns are displayed as mean ± SD using the peak areas detected from 30 pooled larvae (n=3). The *p*-values for M11 in the different groups were calculated by one-way ANOVA (** $p<0.01$, *** $p<0.001$). nq: Confirmed mass, but not quantified due to peak detection below signal-to-noise ratio of 3.

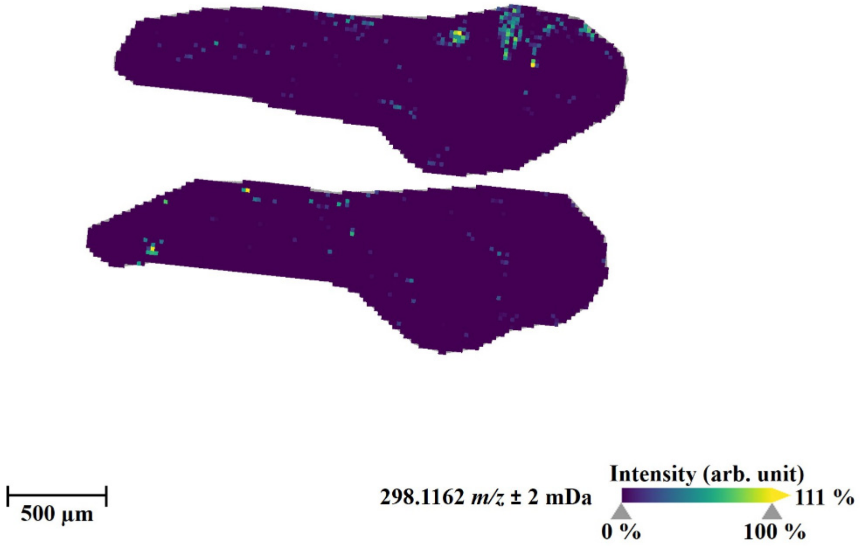

(a)

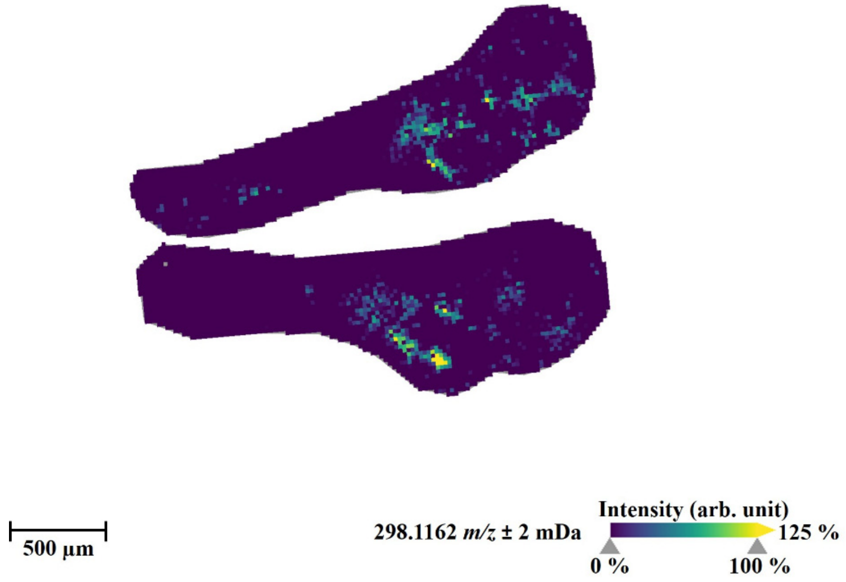

(b)

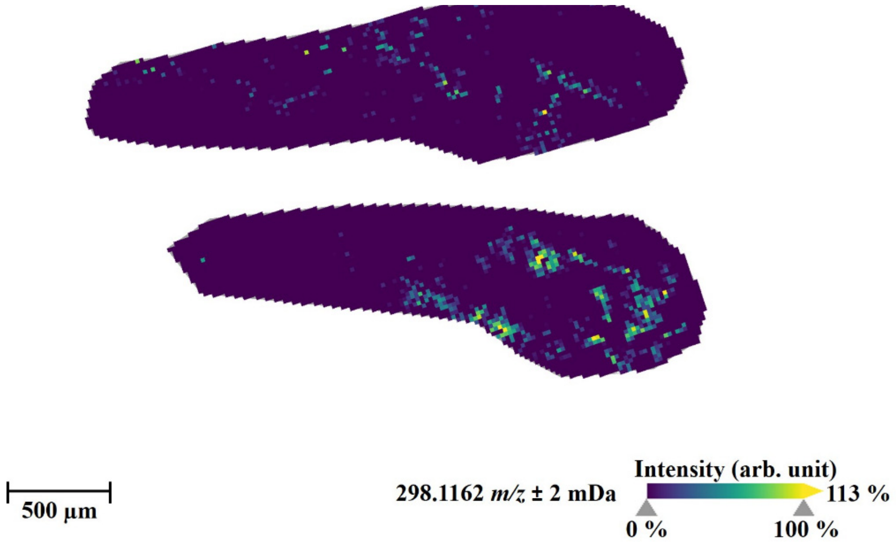

(c)

Figure S2. MALDI-MS images of the most abundant metabolites M3 (ester hydrolysis in combination with *N*-dealkylation, sodium adduct, *m*/*z* 298.1162) in zebrafish larvae at 4 days post-fertilization (dpf) exposed by 4F-MDMB-BINACA for 1 h at 28 °C through direct administration routes via microinjection into caudal vein (a), heart ventricle (b), and hindbrain (c). The presented sections originated from one representative larva per condition. The images were generated by preparing a colormap from blue (no detection) to yellow (high local concentration), and images were further processed in 96 dpi resolution with 24-bit color under no denoising state. Each panel shows two different slices of the same specimen.

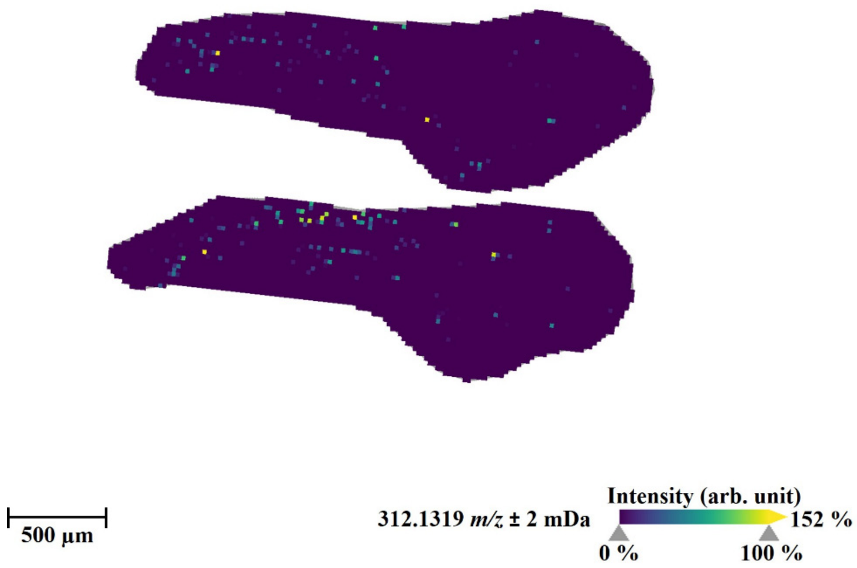

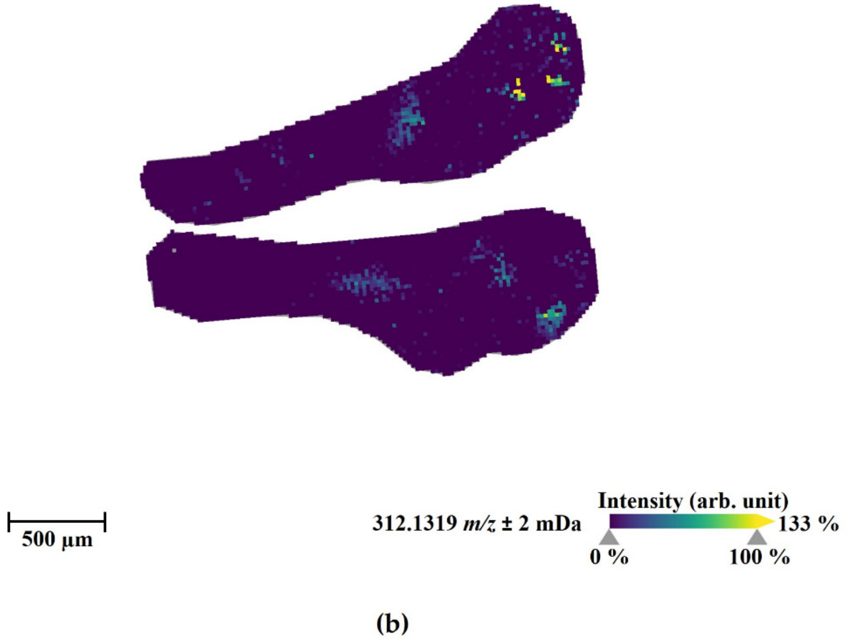

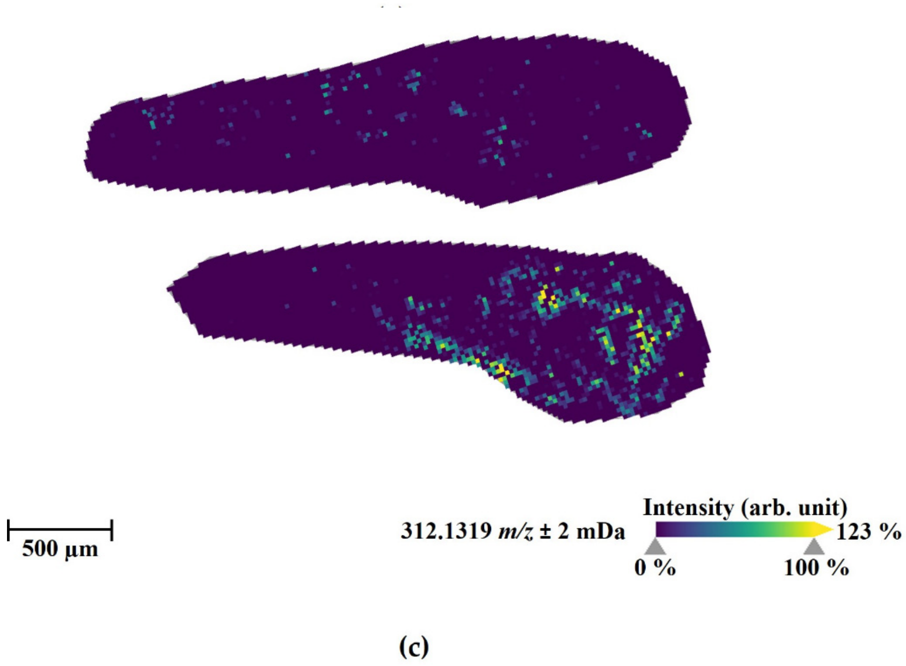

Figure S3. MALDI-MS images of the most abundant metabolite M4 (*N*-dealkylation form, sodium adduct, *m/z* 312.1319) in zebrafish larvae at 4 days post-fertilization (dpf) exposed by 4F-MDMB-BINACA for 1 h at 28 °C through direct administration routes via microinjection into caudal vein (a), heart ventricle (b), and hindbrain (c). The presented sections originated from one representative larva per condition. The images were generated by preparing a colormap from blue (no detection) to yellow (high local concentration), and images were further processed in 96 dpi resolution with 24-bit color under no denoising state. Each panel shows two different slices of the same specimen.

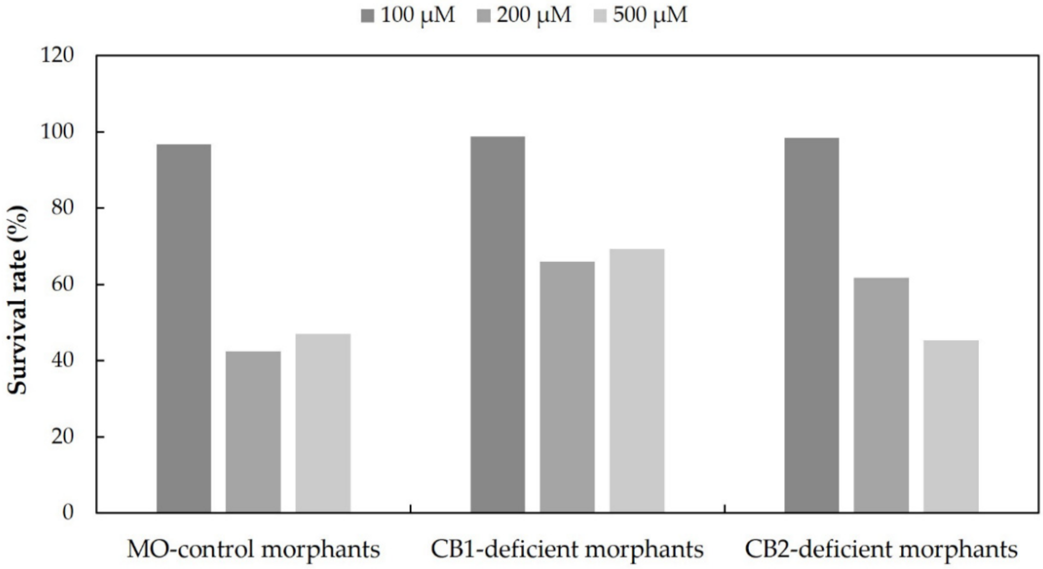

Figure S4. The survival rates of zebrafish mutant larvae at 4 days post-fertilization (dpf) according to the different concentrations of each morpholino oligonucleotide (MO) at 100 μM, 200 μM, and 500 μM with the fixed injection volume of 4.19 nL. All mutant larvae were prepared by 4.19 nL injections of MOs at a one-cell stage. Survival rates (%) were calculated based on the total numbers of ZF mutant larvae measured per condition due to the variance of offspring quality and quantity in parent fish. [n = 126-205 (at 100 μM), 80-136 (at 200 μM), 83-101 (at 500 μM)]. The *p*-values between the 100 μM group showing 100 % survival and the two other groups (200 μM, 500 μM) were computed by one-way ANOVA, and all *p*-values showed significance (*$p<0.05$).

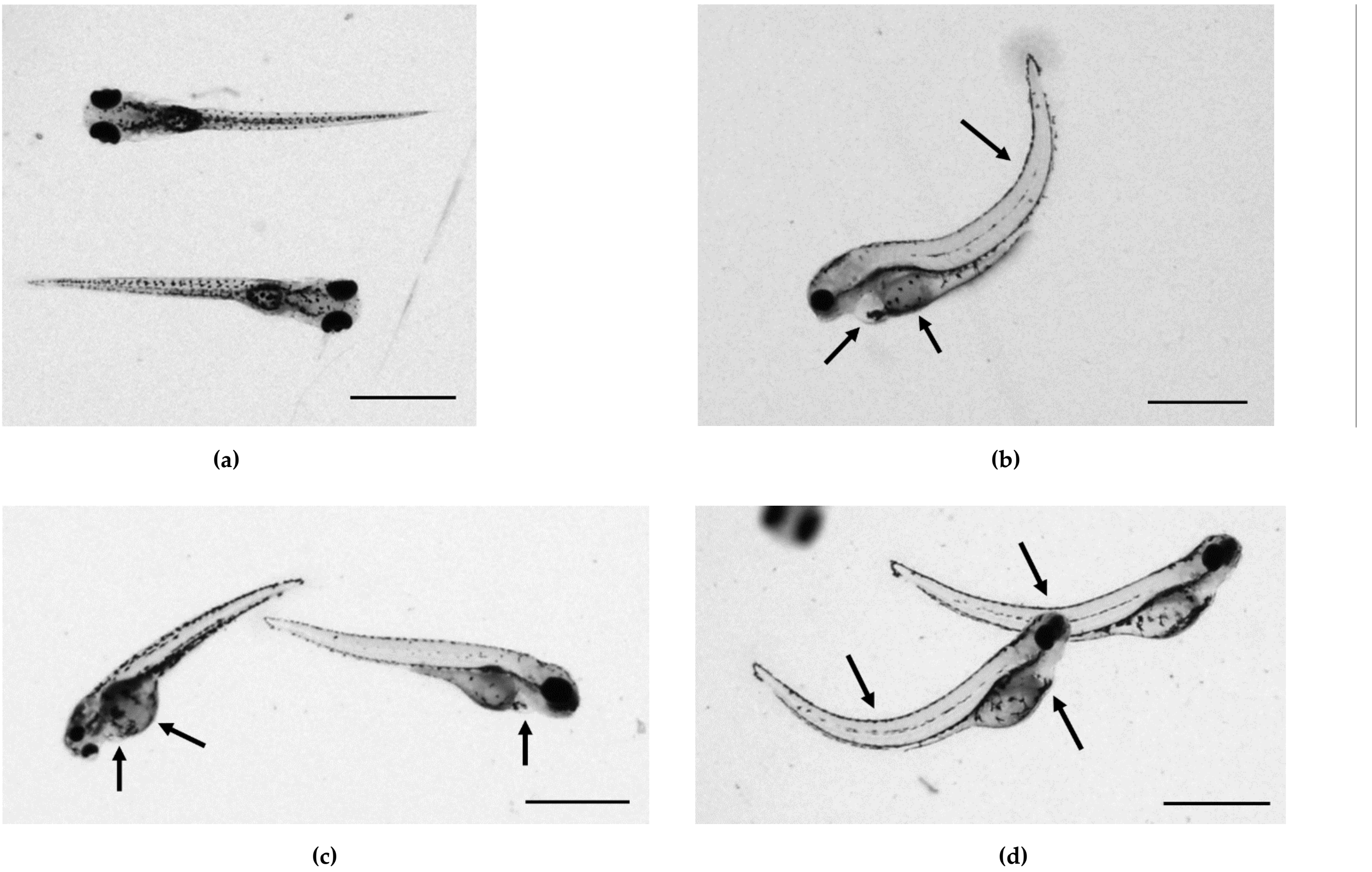

Figure S5. Representative images of morphological malformations and signs of toxicity (depicted by black arrows) in zebrafish larvae at 5 days post-fertilization (dpf) as observed in CB knockdown experiments (MO). The control group, which was left untreated (a), did not show any malformation, whereas some ZF larvae injected with the random MO control 25-*N* (b) displayed edema in the heart ventricle, an up-curved spine, and non-spherical yolk sac. Several CB1 knockdown ZF larvae (c) showed edemas in the heart ventricle and an abnormally large size of the yolk sac. Similarly, several CB2 knockdown ZF larvae (d) showed up-curved spines and heart ventricle edema. The displayed larvae in panel (b)-(d) were created by 4.19 nL injection of each MO (100 μM) at a one-cell stage. To study the impact of MO injection on larval development, all ZF larvae were checked daily under a microscope, and deformed morphants were excluded from the experiments for drug metabolisms studies. Scale bars: 1 mm.

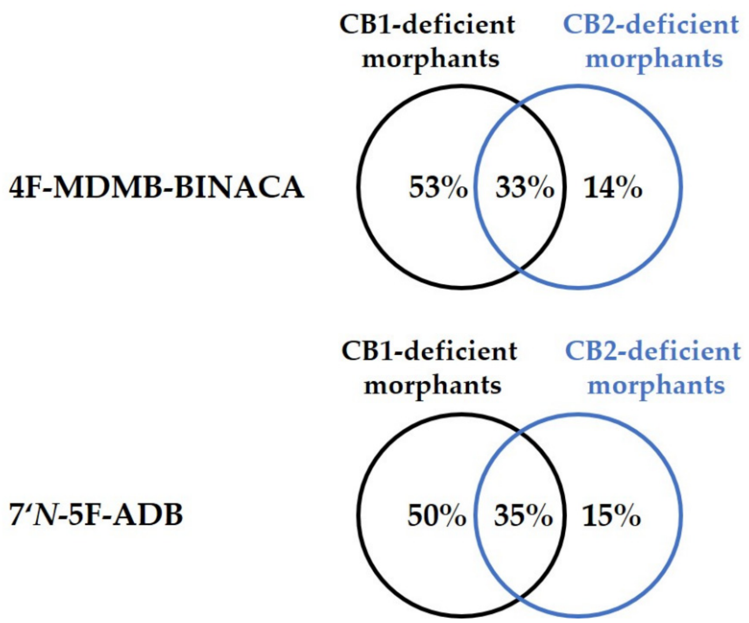

Figure S6. Mutual comparability of the overall metabolites detected in CB1-deficient and CB2-deficient ZF larvae after waterborne exposure of 4F-MDMB-BINACA (at 25 μM) and 7'*N*-5F-ADB (at 50 μM) using Venn diagrams (n=3).

Table S1. Detailed information of 4F-MDMB-BINACA and its phase I and phase II metabolites and their detection in human biosamples from published literature and microinjected zebrafish larvae. All metabolites were identified from three different models in our previous study [27].

Compound	Metabolite ID	Calculated exact masses, m/z	Metabolic reaction	Integrated Human Screening Data* [27,32,34,35]								Data from Zebrafish Larvae			
												Aquatic Exposure, Published Data [27]	Microinjection		
				Plasma [27] (n=1)	Blood [32] (n=4)	Integrated Blood [27,32]	Urine [27] (n=1)	Urine [35] (n=17)	Urine [32] (n=4)	Urine [34] (n=20)	Integrated Urine [27,32,34,35]		Caudal Vein	Heart Ventricle	Hind-brain
Parent compound	4F-MDMB-BINACA	364.2031	Parent Compound	+++			+					+++	+++	+++	+++
Phase I	M1	237.1034	Amide Hydrolysis												
	M2	274.1186	Lactone formation + *N*-dealkylation									+	+nq		+nq
	M3	276.1343	Ester hydrolysis + *N*-dealkylation						+	++	√√		+nq	+nq	+nq
	M4	290.1499	*N*-Dealkylation	+	+	√√						+	+	+	+
	M5	346.1761	Lactone formation + oxidative defluorination									+			
	M6	348.1718	Lactone formation	+	+	√	+	++	+	+	√√	++	+	+	+
	M7	348.1918	Ester hydrolysis + oxidative defluorination					+	+	+	√	+			
	M8	350.1874	Ester hydrolysis	++	++	√	+	+++	+++	+++	√	+			
	M9	360.1554	Lactone formation + oxidative defluorination + oxidation to carboxylic acid									+			
	M10	362.1710	Ester hydrolysis + oxidative defluorination + oxidation to carboxylic acid					+	+	+	√√	+	+nq	+nq	+nq
	M11	362.2074	Oxidative defluorination		+++			+		+	√√	+	+	+	+
	M12	364.1667	Lactone formation + hydroxylation of the tertiary butyl part									+	+	+	++
	M13	366.1824	Ester hydrolysis + hydroxylation of the tertiary butyl part isomer 1				++	+	+		√	+			

	M14	366.1824	Ester hydrolysis + hydroxylation of the indazole part isomer 2					+		+	√				
	M15	376.1867	Oxidative defluorination + oxidation to carboxylic acid					+	++	+	√√	+	+	++	+
	M16	380.1980	Hydroxylation of the tertiary butyl part isomer 1	+				+				+			
	M17	380.1980	Hydroxylation of the butyl chain isomer 2					+							
	M18	380.1980	Hydroxylation of the indazole part isomer 3					+				+			
	M19	380.1980	Hydroxylation of the indazole part isomer 4					+							
Total number of Phase I metabolites				4	4	3	3	12	7	8	9	14	8	7	8
Phase II	M20	460.1548	Hydroxylation of the indazole part + sulfation									+	++	+	+
	M21	524.2239	Ester hydrolysis + oxidative defluorination + glucuronidation												
	M22	526.2196	Ester hydrolysis + glucuronidation				+++								
	M23	542.2144	Ester hydrolysis + hydroxylation of the indazole part + glucuronidation												
	M24	552.2188	Oxidative defluorination + oxidation to carboxylic acid+ glucuronidation									+			
	M25	556.2301	Hydroxylation of the tertiary butyl part + glucuronidation isomer 1									+	+nq	+	+
	M26	556.2301	Hydroxylation of the indazole part + glucuronidation isomer 2									+			
Total number of Phase II metabolites							1					4	2	2	2
Total number of detected Phase I/II metabolites				4	4	3	4	12	7	8	9	18	10	9	10

[1] All human data were integrated with the recently published studies; √: Detection at least twice among the samples in each respective human matrix; √√: detection in both the microinjected ZF larvae and each human biosample; [nq] confirmed mass, but not quantified due to peak detection below signal-to-noise ratio of 3. +: Peak detected, ++: second most abundant peak among metabolites, +++: most abundant peak among metabolites.

Table S2. Mass list of 4F-MDMB-BINACA and its phase I and phase II metabolites used for LC-HRMS/MS and MALDI-FT-ICR measurements.

**Compounds**	**Metabolite ID**	**[M + H]+ ion for LC-HRMS/MS** [27]	**[M + Na]+ ion for MALDI-FT-ICR**
Parent compound	4F-MDMB-BINACA	364.2031	386.1850
Phase I metabolites	M1	237.1034	259.0853
	M2	274.1186	296.1006
	M3	276.1343	298.1162
	M4	290.1499	312.1319
	M5	346.1761	368.1581
	M6	348.1718	370.1537
	M7	348.1918	370.1737
	M8	350.1874	372.1694
	M9	360.1554	383.1452
	M10	362.1710	384.1530
	M11	362.2074	384.1894
	M12	364.1667	386.1487
	M13	366.1824	388.1643
	M14	366.1824	388.1643
	M15	376.1867	398.1686
	M16	380.1980	402.1800
	M17	380.1980	402.1800
	M18	380.1980	402.1800
	M19	380.1980	402.1800
Phase II metabolites	M20	460.1548	482.1368
	M21	524.2239	546.2058
	M22	526.2196	548.2015
	M23	542.2144	564.1964
	M24	552.2188	574.2007
	M25	556.2301	578.2120
	M26	556.2301	578.2120

Table S3. Comparison of the total number of metabolites detected in zebrafish larvae and human urine samples for 7′*N*-5F-ADB and 4F-MDMB-BINACA.

Compound	Total number of metabolites	Human urine samples		Microinjected zebrafish larvae[1]	
		Phase I metabolites	Phase II metabolites	Phase I metabolites	Phase II metabolites
7′*N*-5F-ADB [28]	36	17	10	17 (12)[2]	7 (7)[2]
4F-MDMB-BINACA	26	9[3]	0[3]	8+1[4] (5)[2]	2 (0)[2]

[1] Zebrafish larvae were exposed to the parent compound via microinjection at a final concentration of 5 mM. [2] The values in parentheses stand for the number of overlapping metabolites to human urine samples per compound. [3] The human urine data for 4F-MDMB-BINACA was generated based on literature [27,32,34,35]. [4] The 4F-MDMB-BINACA metabolite M1 detected by MALDI-FT-ICR analysis was included in the ZF larvae data.

Table S4. Number of morphological malformation cases (given in % of total population obeserved) in the zebrafish mutant larvae from 3 days post-fertilization (dpf) to 4 dpf after MOs injection at a one-cell stage with three different concentrations.

	at 3 dpf			at 4 dpf		
	**100 μM**	**200 μM**	**500 μM**	**100 μM**	**200 μM**	**500 μM**
MO-control morphants	11	10	7	11 (11+0)	10 (10+0)	7 (7+0)
CB1-deficient morphants	10	6	14	12 (10+2)	9 (6+3)	17 (14+3)
CB2-deficient morphants	12	15	17	16 (12+4)	15 (15+0)	20 (17+3)

All MOs were injected with a 4.19 nL volume into one-cell stage zebrafish embryos. The defect rates (%) were calculated based on the total sample number due to the difference in offspring quality and quantity used in the experiments. All values in parentheses are % of malformed larvae at 3 dpf + % of malformed larvae at 4 dpf. [n = 126-205 (at 100 μM), n = 80-136 (at 200 μM), n = 83-101 (at 500 μM)].

Table S5. Sequences of morpholino oligonucleotides (MOs) for the gene knockdown of CB1 and CB2 by binding its splicing site.

Gene	Morpholino sequence
cnr1	GTGCTATCAACAACATACCTTTGTG[a]
cnr2	GCCATGAAACAAACAGTACCTGTGG[a]
n.a. (random control 25-N)	NNNNNNNNNNNNNNNNNNNNNNNNN

[a] The sequences of MOs for CB1 and CB2 were validated in earlier studies [38,58].
